# Prostate Health Index and Multiparametric MRI: Partners in Crime Fighting Overdiagnosis and Overtreatment in Prostate Cancer

**DOI:** 10.3390/cancers13184723

**Published:** 2021-09-21

**Authors:** Matteo Ferro, Felice Crocetto, Dario Bruzzese, Massimo Imbriaco, Ferdinando Fusco, Nicola Longo, Luigi Napolitano, Evelina La Civita, Michele Cennamo, Antonietta Liotti, Manuela Lecce, Gianluca Russo, Luigi Insabato, Ciro Imbimbo, Daniela Terracciano

**Affiliations:** 1 Division of Urology, European Institute of Oncology (IEO), IRCCS, 20141 Milan, Italy; matteo.ferro@ieo.it; 2 Department of Neurosciences, Reproductive Sciences and Odontostomatology, University of Naples “Federico II”, 80131 Naples, Italy; felice.crocetto@unina.it (F.C.); nicola.longo@unina.it (N.L.); luigi.napolitano12@studenti.unina.it (L.N.); ciro.imbimbo@unina.it (C.I.); 3 Department of Public Health, University of Naples “Federico II”, 80131 Naples, Italy; dario.bruzzese@unina.it (D.B.); gianlucar93@libero.it (G.R.); 4 Department of Advanced Biomedical Sciences, University of Naples “Federico II”, 80131 Naples, Italy; massimo.imbriaco@unina.it (M.I.); luigi.insabato@unina.it (L.I.); 5 Department of Woman, Child and General and Specialized Surgery, University of Campania Luigi Vanvitelli, 80138 Naples, Italy; ferdinando-fusco@libero.it; 6 Department of Translational Medical Sciences, University of Naples “Federico II”, 80131 Naples, Italy; e.lacivita@studenti.unina.it (E.L.C.); michele.cennamo2@unina.it (M.C.); antonietta.liotti@unina.it (A.L.); m.lecce@studenti.unina.it (M.L.)

**Keywords:** prostate cancer, multiparametric magnetic resonance imaging, PHI, PHI density, biopsy

## Abstract

**Simple Summary:**

In the last decades, the widespread use of PSA as the standard tool for prostate cancer diagnosis led to a high rate of overdiagnosis and overtreatment. More recently, multiparametric magnetic resonance imaging (mpMRI) became part of the diagnostic pathway, and several next-generation PSA-based tests (PHI, PHI density, 4Kscore, STHLM3) have been proposed. The multivariable approach promises to help with a better stratification of PCa patients at initial diagnosis. In this study, we evaluated the performance of the prostate health index (PHI) and mpMRI for the prediction of positive biopsy and of high-grade PCa at radical prostatectomy (RP). Our findings suggested that PHI had a better ability than mpMRI to predict positive biopsy, whereas a comparable performance in the identification of pathological aggressive PCa was pointed out. Notably, PHI and PHI density might represent useful biomarkers to recognize high-grade PCa in patients with low or uncertain PI-RADS scores on mpMRI.

**Abstract:**

Widespread use of PSA as the standard tool for prostate cancer (PCa) diagnosis led to a high rate of overdiagnosis and overtreatment. In this study, we evaluated the performance of the prostate health index (PHI) and multiparametric magnetic resonance imaging (mpMRI) for the prediction of positive biopsy and of high-grade PCa at radical prostatectomy (RP). To this end, we prospectively enrolled 196 biopsy-naïve patients who underwent mpMRI. A subgroup of 116 subjects with biopsy-proven PCa underwent surgery. We found that PHI significantly outperformed both PI-RADS score (difference in AUC: 0.14; *p* < 0.001) and PHI density (difference in AUC: 0.08; *p* = 0.002) in the ability to predict positive biopsy with a cut-off value of 42.7 as the best threshold. Conversely, comparing the performance in the identification of clinically significant prostate cancer (csPCa) at RP, we found that PHI ≥ 61.68 and PI-RADS score ≥ 4 were able to identify csPCa (Gleason score ≥ 7 (3 + 4)) both alone and added to a base model including age, PSA, fPSA-to-tPSA ratio and prostate volume. In conclusion, PHI had a better ability than PI-RADS score to predict positive biopsy, whereas it had a comparable performance in the identification of pathological csPCa.

## 1. Introduction

Multiparametric magnetic resonance imaging (mpMRI) of the prostate currently plays a central role in the diagnostic pathway of suspected prostate cancer (PCa) [1] and is among the gold standards for the prediction of positive biopsy [2]. The Prostate Imaging Reporting and Data System version 2 (PI-RADS v2) had allowed for reliable identification of clinically significant (cs) PCa needing biopsy and helped direct targeting of lesions [3,4]. Men who have a PI-RADS score of 3 or higher underwent biopsy [5]. However, PI-RADS 3 corresponds to csPCa only in less than 15% of patients [1]. Thus, a score of 3 represents a “gray zone” in mpMRI with low rates of csPCa, and there is an urgent need to have tools able to avoid unnecessary biopsies without missing aggressive cancers. Moreover, positive predictive value (PPV) was 0.49 for csPCa, and a few patients with a negative mpMRI had high-grade PCa [5]. Thus, the use of mpMRI to select patients for biopsy is not ideal [6,7,8]. The use of biomarkers could aid in avoiding unnecessary biopsies without missing aggressive cancers [9]. In this scenario, a pivotal role is played by the prostate health index (PHI), which showed a good ability to identify csPCa [10]. Several reports indicated that the use of PHI in clinical routine, when compared with total and free PSA, produced a significant decrease in unnecessary biopsies and a reduction in the percentage of low-risk diagnosed PCa [11,12,13,14,15,16,17,18]. However, only few authors investigated the predictive performance of PHI combined and compared with mpMRI [19,20] and how PHI performs in patients stratified by PI-RADS score [21,22,23]. Interestingly, some authors analyzed the ability of the combination of PHI density (the ratio between PHI value and prostate volume) and mpMRI to identify high-grade PCa [24]. Moreover, the combination of PHI and mpMRI has been investigated as a tool to predict grade reclassification in men on active surveillance (AS). Such an approach may be useful to decrease the frequency of surveillance prostate biopsies, ultimately leading to improved patient compliance with AS protocols, considerable improvements in patient quality of life and reduction in treatment costs associated with AS [25]. Therefore, further additions to the growing literature in this area are still needed. To this end, we evaluated the performance of the PHI, PHI density and mpMRI in the prediction of positive biopsy. In addition, we compared the ability of these three variables to identify surgical high-grade PCa. Finally, we correlated PHI and PHI density with PI-RADS score and evaluated the ability of biomarkers to recognize csPCa in patients with PI-RADS score ≤ 3.

## 2. Materials and Methods

### 2.1. Study Design

We performed a monocentric, prospective and observational study between May 2020 and July 2021 to assess whether PHI can improve the identification of csPCa in patients who underwent mpMRI. Patients underwent a standard prostatic biopsy, which consisted of at least 16 needle biopsy cores obtained under transrectal ultrasound (TRUS) guidance.

Patients with PI-RADS ≥ 3 received a targeted biopsy with 12-core standard systematic biopsy. PSA values included between 2 and 10 ng/mL and/or a suspicious digital rectal exploration without a previous prostate biopsy. Subjects suffering from acute prostatitis or urinary tract infections, subjects with previous surgery or biopsy and subjects using 5-α reductase inhibitors were excluded. Prostate volume was calculated by transrectal ultrasound (TRUS).

Primary and secondary Gleason scores were assigned by a single genitourinary pathologist blinded to the biomarker values, according to the 2005 consensus conference of the International Society of Urological Pathology definitions [26].

Among the 196 enrolled patients, 116 with biopsy-proven PCa underwent robot-assisted radical prostatectomy (RALP) at the Division of Urology of the University Federico II (Naples, Italy) within 3 months. None of the study patients received neoadjuvant hormonal therapy (antiandrogens or luteinizing hormone-releasing hormone analogs or antagonists) and/or other drugs (i.e., 5-α reductase inhibitors) that could alter the PSA values. For the definition of csPCa, we used postoperative Gleason score ≥ 7 (4 + 3) [27,28]. Informed consent was obtained from every study participant before the surgical procedure. The study was conducted in accordance with the Declaration of Helsinki, and the protocol was approved by the Ethics Committee of the University of Naples “Federico II” (project identification code 118/20).

### 2.2. Biomarker Measurement

Participants had blood drawn before DRE at each visit. Whole blood was allowed to clot before serum was separated by centrifugation. Serum aliquots were stored at −80 °C until samples were processed, according to Semjonow et al. [29]. Specimens were analyzed in blinded fashion for PSA, fPSA and p2PSA by Access2 Immunoassay System analyzer (Beckman Coulter, Brea, CA, USA) calibrated against the WHO standard for PSA and fPSA. The analytical performance of the measurements assessed with control materials (Beckman Coulter) showed values within the allowed recommended limits. PHI density was calculated as the ratio between PHI value and prostate volume.

### 2.3. Multiparametric MRI and Biopsy Protocols

mpMRI was performed using a 3-T MRI scanner Siemens MAGNETOM Vida 3 T (Siemens, Munich, Germany) acquiring diffusion-weighted imaging (DWI), dynamic contrast enhancement imaging (DCE), T1-weighted axial and T2-weighted triplanar imaging. All mpMRI images were independently interpreted by four different experienced genitourinary radiologists, with at least 5 years of experience, according to PI-RADS version 2.0. The images were segmented to obtain and record lesion locations and PI-RADS scores.

### 2.4. Statistical Analysis

Standard descriptive statistics were used to characterize the overall sample: mean ± standard deviation or median [25th percentile; 75th percentile] with range in case of numerical variables and absolute frequencies with percentages in case of categorical factors. Between-group differences were evaluated using *t*-test for independent samples, the nonparametric Mann–Whitney test, the chi-square test or the Fisher test where appropriate. Discrimination ability of the different PSA-derived biomarkers, as well as PI-RADS score, was assessed using ROC curve and further quantified computing the area under the curve (AUC) with the corresponding 95% confidence interval (95% CI). Optimal thresholds were defined as those maximizing the Youden index (sensitivity + specificity − 1). Multivariable logistic regression models were built to assess the independent and incremental capacity of PHI, PHI density and PI-RADS score in identifying PCa and csPCa with respect to a set of base clinical variables including age, PSA, fPSA-to-tPSA ratio and prostate volume. All statistical comparisons involving two AUCs, as well as the estimation of the 95% CIs of sensitivity and specificity associated with the optimal thresholds, were based on bootstrap resampling. Statistical analyses were performed using the R language (vers. 4.0.3, R Foundation for Statistical Computing, Vienna, Austria). *p*-values < 0.05 were considered statistically significant.

## 3. Results

The overall cohort consisted of 196 patients with a mean age of 66.6 ± 7.8 years (range: 48 to 85 years). Mean PSA was equal to 6.2 ± 2 ng/mL (range: 2 to 10 ng/mL); PI-RADS score was equal to or greater than 4 in 111 patients (56.6%) and equal to 3 in 44 patients (16.8%). Median PHI was equal to 51.1 (range: 12.7 to 179.3) (Table 1).

### 3.1. Biopsy Outcome

**Figure 1:**
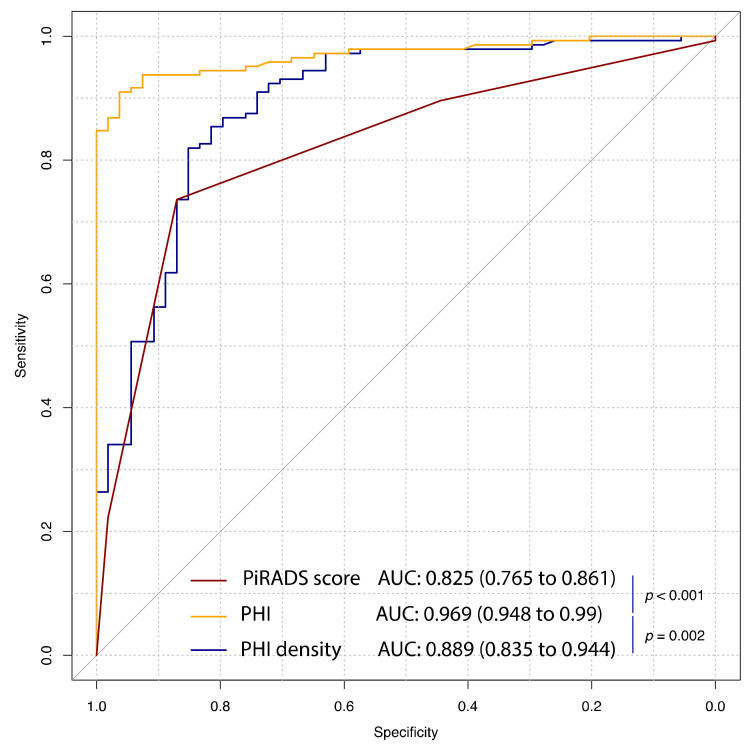
Receiver operating characteristic (ROC) curve analysis for PHI, PHI density and mpMRI to predict PCa at first biopsy. Solid black line refers to the useless classifier in which the false positive rate (1 − specificity) equals the true positive rate (sensitivity). PiRADS, Prostate Imaging Reporting and Data System; PHI, prostate health index; AUC, area under curve; mpMRI, multiparametric magnetic resonance; PCa, prostate cancer.

In the diagnostic setting, a positive biopsy was observed in 142 patients (72.4%). All PSA-derived biomarkers, as well as PI-RADS score, were significantly associated with the risk of PCa at univariate analysis with an AUC ranging from 0.65 (95% CI: 0.559 to 0.742) for free PSA to 0.969 (95% CI: 0.948 to 0.990) in case of PHI (Table 2). In the head-to-head comparison, PHI significantly outperforms, in terms of diagnostic accuracy, both PI-RADS score (difference in AUC: 0.14; *p* < 0.001) and PHI density (difference in AUC: 0.08; *p* = 0.002) (Figure 1). The optimal cut-off was 42.7 for PHI and was associated with a sensitivity equal to 90.8% (95% CI: 85.9% to 95.1%) and a specificity equal to 96.3% (95% CI: 90.7% to 100%). By using a PI-RADS score equal to or greater than 4, a sensitivity of 74.6% (95% CI: 67.6% to 81.7%) and a specificity of 87% (95% CI: 77.8% to 94.4%) was reached. The best threshold for PHI density was equal to 0.781 and was associated with a sensitivity of 81.7% (95% CI: 74.6% to 88%) and a specificity of 85.2% (95% CI: 75.9% to 94.4%) (Table 2).

### 3.2. Radical Prostatectomy Outcome

Among the 142 patients with positive biopsy, prostatectomy data were available for a subset of 116 patients (81.7%) with a mean age of 65.6 ± 6.9 years (range: 48 to 81) (Table 1).

**Figure 2:**
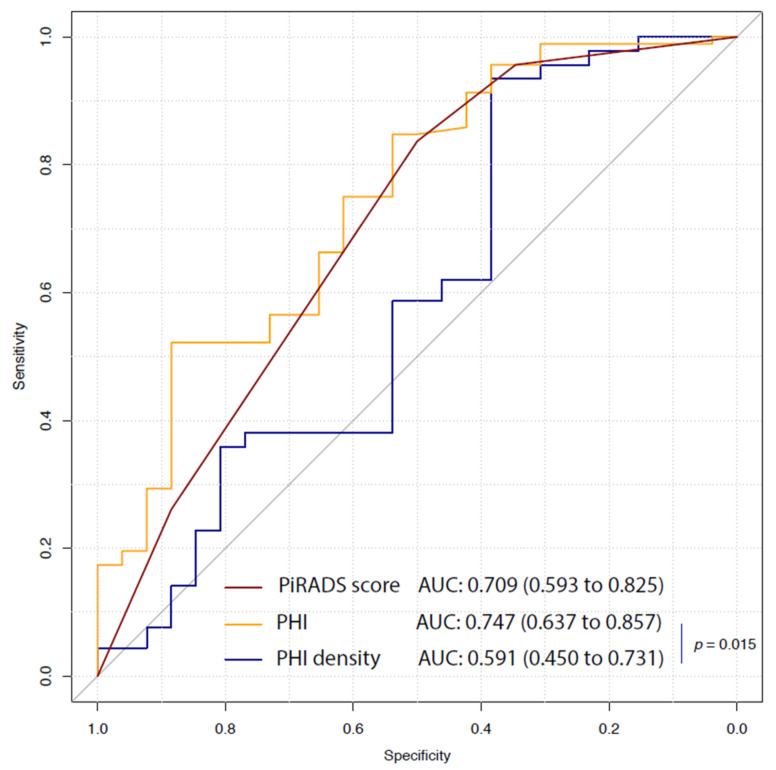
Receiver operating characteristic (ROC) curve analysis for PHI, PHI density and mpMRI to predict csPCa at RP. Solid black line refers to the useless classifier in which the false positive rate (1 − specificity) equals the true positive rate (sensitivity). PiRADS, Prostate Imaging Reporting and Data System; PHI, prostate health index; AUC, area under curve; mpMRI, multiparametric magnetic resonance; csPCa, clinically significant prostate cancer; RP, radical prostatectomy.

A clinically significant PCa (Gleason score 7 (3 + 4) or higher) was observed in 90 patients (77.6%). Only PHI (as well as the ratio between p2PSA and free PSA) and PI-RADS score, but not PHI density, preserved a significant association with the presence of csPCa at surgery with an AUC equal, respectively, to 0.747 (95% CI: 0.637 to 0.857) and 0.709 (95% CI: 0.593 to 0.825) (Figure 2). However, the difference between PHI and PI-RADS score was no longer statistically significant (difference in the AUC: 0.038 *p* = 0.536). The optimal cut-off for the identification of csPCa at prostatectomy remained unchanged for PI-RADS (4; sensitivity: 85.6%, 95% CI: 77.8 to 92.2; specificity: 50%, 95% CI: 30.8 to 69.2), whereas it became higher for PHI (61.68; sensitivity: 53.3%, 95% CI: 43.3 to 63.3; specificity: 88.5%, 95% CI: 76.9 to 100) (Table 3).

Both PI-RADS and PHI allowed improvement in the diagnostic accuracy with respect to a base model based on age, PSA, fPSA-to-tPSA ratio and prostate volume, characterized by an AUC of 0.684 (95% CI: 0.0.562 to 0.806). By adding PHI to the set of base variables, the AUC of the model was raised to 0.784 (95% CI: 0.676 to 0.891; *p* = 0.044 with respect to the base model); by adding PI-RADS to the base variables, an almost equal increase was observed (AUC: 0.786; 95% CI: 0.66 to 0.912; *p* = 0.026 with respect to the base model) (Table 4).

### 3.3. Correlation between PHI and PI-RADS Score

**Figure 3:**
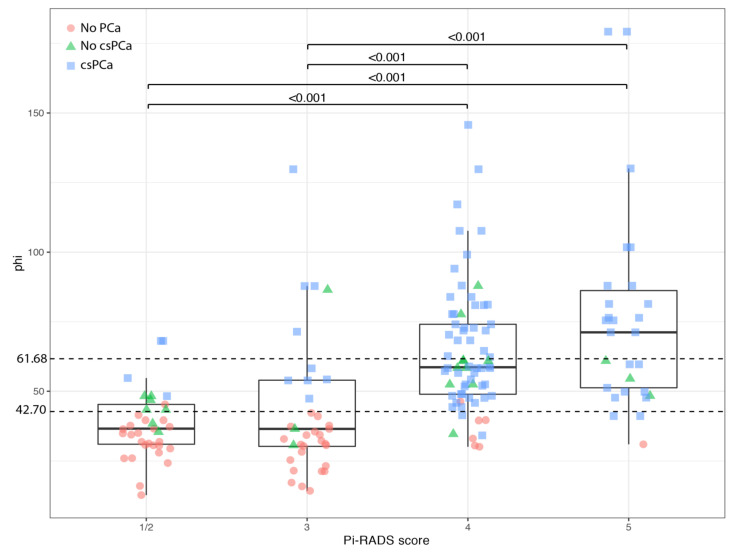
Boxplot showing distribution of PHI according to PI-RADS v2 score. Dashed lines indicate cut-off values. Data are shown as median (bold horizontal line in the box) and Q1 and Q3 (borders of the box). Whiskers represent the lowest and the highest values that are not outliers (i.e., data points below Q1 − 1.5 × IQR or above Q3 + 1.5 × IQR). Outliers are those outside the whiskers. Q1 = 25th percentile; Q3 = 75th percentile; IQR (interquartile range) = Q3 − Q1; csPCa, clinically significant prostate cancer; PI-RADS, Prostate Imaging Reporting and Data System; PHI, prostate health index.

**Figure 4:**
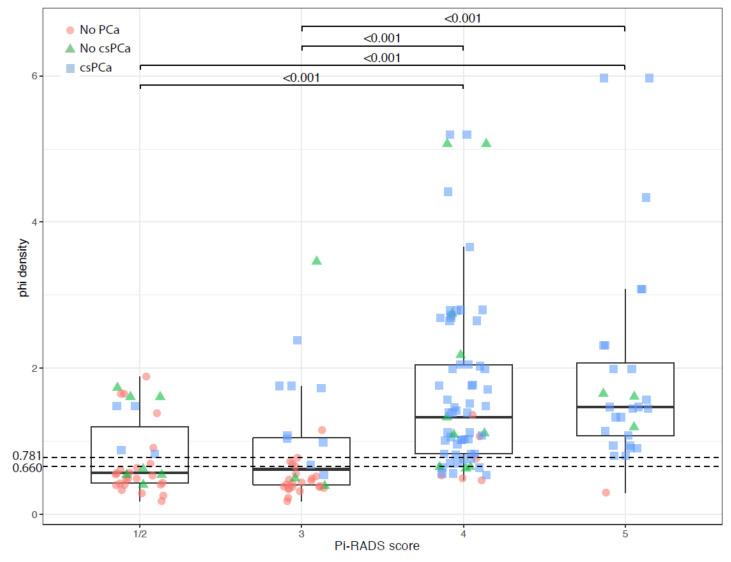
Boxplot showing distribution of PHI density according to PI-RADS v2 score. Dashed lines indicate cut-off values. Data are shown as median (bold horizontal line in the box) and Q1 and Q3 (borders of the box). Whiskers represent the lowest and the highest values that are not outliers (i.e., data points below Q1 − 1.5 × IQR or above Q3 + 1.5 × IQR). Outliers are those outside the whiskers. Q1 = 25th percentile; Q3 = 75th percentile; IQR (interquartile range) = Q3 − Q1; csPCa, clinically significant prostate cancer; PI-RADS, Prostate Imaging Reporting and Data System; PHI, prostate health index.

In the overall cohort of 196 patients, both PHI and PHI density showed significant differences among the classes of PI-RADS score. Patients with PI-RADS score ≤ 2 and subjects with PI-RADS equal to 3 presented significantly lower PHI values than patients with a PI-RADS score equal to 4 or 5 (Figure 3). An almost equal pattern was observed for PHI density (Figure 4).

In addition, PHI values higher than 42.7 (Figure 3) and PHI density higher than 0.660 (Figure 4) were associated with the presence of csPCa in subjects with PI-RADS score ≤ 3.

## 4. Discussion

Novel biomarkers and MRI are well-known tools useful to identify csPCa [19,20,22]. To the best of our knowledge, this study is the largest cohort study (*n* = 196) centered on the evaluation of PHI and mpMRI findings according to PI-RADS version 2, which included not only patients who underwent biopsy but also a subgroup (*n* = 116) of subjects treated by RP. Thus, our study population also allowed us to evaluate the ability to recognize the presence of pathological csPCa.

We found that PHI significantly outperformed both mpMRI and PHI density in the prediction of positive biopsy. A cut-off value of 42.7 has been identified as the best threshold. In addition, we compared the ability of PHI, PHI density and mpMRI to predict the presence of csPCa at RP. We found that PHI ≥ 61.68 and PI-RADS score ≥ 4 were able to identify csPCa (Gleason score ≥ 7 (3 + 4)) both alone and added to a base model including age, PSA, fPSA-to-tPSA ratio and prostate volume. We also observed that p2PSA/fPSA, but not PHI density, had the same ability as PHI in the recognition of csPCa. Finally, we demonstrated a good correlation of PHI and PHI density with PI-RADS score. Of note, increased PHI and PHI density values allowed identifying csPCa in subjects with PI-RADS score ≤ 3 corresponding to negative or uncertain mpMRI outcomes. Our findings suggested that PHI and mpMRI showed a comparable ability to predict csPCa defined at RP. A sequential or combined use of the test and imaging might ensure the best ability to reduce unnecessary biopsies without missing aggressive PCa as previously demonstrated by other authors. In the first report of the complementary role of PHI and mpMRI in repeated biopsies [19], the authors found that the addition of PHI to mpMRI provided an improvement in the prediction of both overall cancers and csPCa with AUCs respectively of 0.71 and 0.75 compared to mpMRI (AUC 0.64) and PSA alone (AUC 0.69). Decision curve analysis showed that a PHI cut-off ≥ 35 in patients with negative mpMRI corresponded to a negative predictive value of 0.97 for the absence of csPCa. Accordingly, in a prospective Asian cohort including candidates for first biopsy, Hsieh et al. showed an AUC of 0.87 for the PHI combined with mpMRI [20]. The authors also reported that if biopsies were limited to subjects with PHI values ≥ 30 and PI-RADS score ≥ 3, it was possible to save about 50% of unnecessary biopsies, missing only one csPCa. In a prospective study on 345 patients at Johns Hopkins University, Tosoian et al. found that PI-RADS score ≤ 3 and PHI levels < 27 corresponded to the absence of csPCa in 15 men on first biopsy [21]. Druskin et al. obtained a similar result for PHI density, which was able to distinguish csPCa in men with PI-RADS 1 (negative MRI) and PI-RADS 3 (uncertain MRI) [24]. These findings agree with our results and support the hypothesis that PHI may be a useful tool to recognize high-grade PCa beyond MRI outcome. Recently, Fan et al. [22] demonstrated that PHI, among PSA-derivative biomarkers, was the best predictor of csPCa in men with PI-RADS score 3 and 4/5. These findings suggested that in patients with PI-RADS 3 index lesions, which is a gray zone for PI-RADS v2, PHI may help to identify high-risk groups for csPCa and may enable several patients to avoid unnecessary biopsy. Accordingly, in a previous report, Tan et al. showed that a PHI cutoff value of ≥ 27 would have allowed 34% of the patients with PI-RADS 3 lesions (*n* = 35) to avoid a targeted biopsy, with both sensitivity and NPV of 100% [23]. In a study published by Stejskal et al. [30] including 395 men, the authors performed a head-to-head comparison between PHI and mpMRI (using the PI-RADS version 1, 1.5 T endorectal coil and 3 T machines), reporting that PHI achieved more accurate prediction for csPCa both in the first (*n* = 249) and repeated (*n* = 144) biopsy subgroups. The authors also showed that adding PHI to PI-RADS significantly increased the accuracy for the prediction of any cancer and csPCa in both the subgroups. Recently, Kim et al. [31] showed that a model for a hypothetical cohort of 1000 patients with elevated PSA using PHI with a cut-off ≥ 30 as a triage test could save both MRI and biopsies by 25% missing the identification of csPCa in a percentage lower than 10% and reducing the cost per referred patient by about 20%.

The proportion of men who harbor csPCa was very different in PI-RADS 3 and in PI-RADS 4/5 [1]. PI-RADS 3 lesions corresponded to high-grade PCa in 16–21% [32].

A low number of csPCa cases remained undiagnosed by negative mpMRI. PI-RADS 1–2 lesions corresponded to about 1 in 10 probability of diagnosis of csPCa [33]. The results of the PROMIS cohort indicated that mpMRI missed cancers of lower grade and often smaller disease compared to detected PCa [34]. In our study cohort, we had a small group of patients with PI-RADS 1–2 and positive biopsy. These patients were subjected to RP and were diagnosed with PCa, according to previously reported mpMRI negative predictive value [33]. Notably, our results suggested that PHI and PHI density allowed detecting csPCa in those subjects. At present, a growing number of patients opt for focal therapy or AS, and thus the accuracy of risk stratification at initial diagnosis is clinically relevant. Further studies are encouraged to characterize the false-negative PI-RADS cancers and to identify tools able to reveal csPCa in subjects with negative mpMRI. In such a context, a biomarker is strongly needed to recognize patients who harbor high-risk PCa and apply a personalized biopsy schedule. This approach may avoid a lot of unnecessary biopsies and definitive treatments with their related well-known side effects. Accordingly, PHI appeared in the diagnostic algorithm for patients with suspected PCa both to select patients for MRI and biopsy [35]. In addition, PHI has been proposed as a tool to select and monitor patients on active surveillance (AS) [36,37,38], and the diagnostic accuracy of PHI for the identification of csPCa might be increased when combined with mpMRI. Several authors showed that the addition of PHI and PHI density to a multivariable model including PI-RADS v2 was able to predict biopsy reclassification with AUCs higher than 0.80 [24,25]. Thus, the combination of PHI and mpMRI may be useful for decreasing the frequency of surveillance prostate biopsies, ultimately leading to (i) improved patient compliance with AS protocols, (ii) considerable improvements in patient quality of life and (iii) reduction in treatment costs associated with AS.

Dall’Era [36] suggested that beyond PSA kinetics, PSA density, [−2]proPSA, PHI, 4k score and Stockholm3 are liquid biomarkers associated with disease reclassification. However, among these last tests, PHI was the cheapest, easier to perform and the only one FDA-approved and CE-marked [10].

We recently demonstrated that by using artificial neuronal networks (ANNs), it is possible to develop models combining different PSA-derivatives (including total PSA, free PSA, p2PSA and PSA density) optimizing high-grade PCa recognition [39]. Taking into account that AI is revolutionizing PCa clinical management [40,41] and ameliorates accuracy in the detection of csPCa when applied to MRI [42], it is plausible to hypothesize that the sequential or the combined use of novel biomarkers and MRI based on machine learning approach may produce models able to minimize overdiagnosis, without missing csPCa, providing the clinicians a tool to match tumor aggressiveness and treatment invasiveness.

To the best of our knowledge, this study was the largest prospective cohort study aiming to evaluate PHI and imaging as partners in crime in the battle against overdiagnosis and overtreatment, which included not only a biopsy-naïve patient setting but also a subgroup that underwent surgery. Thus, one strength of this study is the definition of csPCa based on pathological grading, whereas one limitation was that our results may not be extended to repeated biopsy. Another limitation is that the subgroup of PI-RADS 3 was small, and the results need further studies on a larger number of patients with uncertain mpMRI outcomes, since the role of PHI in this group is a relevant issue to reduce unnecessary biopsies due to mpMRI failure. In addition, a major limitation of this study is that we did not evaluate the results of the follow-up of patients with high PI-RADS score and negative biopsy. This issue is clinically relevant, and thus we are currently collecting the data for a future study.

## 5. Conclusions

In conclusion, our findings suggested that PHI had a very good accuracy as a noninvasive biomarker to predict positive biopsy. PHI and mpMRI had a comparable performance in the recognition of pathological csPCa. PHI and PHI density were able to improve pathological csPCa identification in patients with negative or uncertain mpMRI outcomes. The implementation of PHI in addition to mpMRI in clinical practice may ensure a personalized therapeutic approach for patients with suspected PCa and may bridge the gap of PI-RADS 3 mpMRI outcomes.

## Figures and Tables

**Table 1:** Demographic and clinical characteristics of the study population. Data are reported as mean ± standard deviation (min to max), median [25th percentile; 75th percentile] (min to max) or absolute frequency (percentage).

Parameter	Overall Cohort At Biopsy (*n* = 196)	Overall Cohort At RP (*n* = 116)
Age	66.6 ± 7.8 (48 to 85)	65.6 ± 6.9 (48 to 81)
PI-RADS score		
1/2	39 (19.9)	13 (11.2)
3	44 (22.4)	13 (11.2)
4	80 (40.8)	63 (54.3)
5	33 (16.8)	27 (23.3)
Prostate volume (mL)	51.8 [34.8; 68] (12 to 148)	46 [32; 62] (12 to 120)
PSA (ng/mL)	6.2 ± 2 (2 to 10.4)	6.5 ± 2 (2.6 to 10.4)
PSA density	12 [8; 16.8] (3.3 to 76)	13 [9; 20.2] (4 to 76)
fPSA (ng/mL)	0.81 [0.58; 1.2] (0.15 to 2.45)	0.72 [0.56; 1.12] (0.17 to 2.27)
fPSA/tPSA ratio	14.5 [10.7; 20.4] (3.8 to 44.3)	12.64 [8.6; 17.2] (3.8 to 34.6)
p2PSA (ng/mL)	17.6 [13.2; 25.8] (1.3 to 55.2)	18.3 [13.7; 27.9] (3.29 to 55.2)
p2PSA/fPSA ratio	1.24 [0.87; 1.84] (0.19 to 4.64)	1.48 [1.13; 2.12] (0.7 to 4.64)
PHI	51.1 [37.1; 70.1] (12.7 to 179.3)	58.9 [48.4; 77.7] (30.7 to 179.3)
PHI density	1.03 [0.6; 1.66] (0.18 to 6.61)	1.44 [0.9; 2] (0.39 to 5.97)

PSA, prostate specific antigen; fPSA/tPSA, free PSA/total PSA; p2PSA, [−2]proPSA; PI-RADS, Prostate Imaging Reporting and Data System; PHI, prostate health index; RP, radical prostatectomy.

**Table 2:** Demographic and clinical characteristics of the study population stratified by diagnosis of PCa at biopsy. For each variable, the AUC of the corresponding ROC curve with the 95% confidence interval (CI) is shown. Best threshold for the diagnosis of PCa, according to the maximization of the Youden index, is reported with the accomplished sensitivity and specificity. Cases are defined as subjects with values equal to or greater than threshold, except for prostate volume where the opposite applies.

Studied Variables	No PCa (*n* = 54; 27.3%)	PCa (*n* = 142; 72.7%)	*p*-Value	AUC (95% CI)	Best Threshold	Specificity (95% CI)	Sensitivity (95% CI)
Age	65.5 ± 9 (48 to 85)	67 ± 7.3 (49 to 85)	0.283	-			
PI-RADS score	3 [2; 3] (2 to 5)	4 [3; 4] (1 to 5)	<0.001	0.825 (0.765 to 0.861)	4	87 (77.8 to 94.4)	74.6 (67.6 to 81.7)
Prostate volume (mL)	61.5 [48.8; 78] (24 to 120)	48 [33; 62] (12 to 148)	<0.001	0.666 (0.578 to 0.753)	51.8	74.1 (61.1 to 85.2)	59.2 (50.7 to 66.9)
PSA (ng/mL)	5.4 ± 2 (2 to 9.6)	6.5 ± 1.9 (2.6 to 10.4)	0.001	0.651 (0.563 to 0.739)	4.745	48.1 (35.2 to 61.1)	78.9 (71.8 to 85.2)
PSA density	8.1 [6.1; 12.3] (3.8 to 21.0)	13.0 [9; 19.2] (3.3 to 76)	<0.001	0.743 (0.668 to 0.818)	8.95	59.3 (46.3 to 72.2)	80.3 (73.9 to 86.6)
fPSA (ng/mL)	1.12 [0.7; 1.47] (0.15 to 2.45)	0.75 [0.57; 1.12] (0.24 to 2.27)	0.001	0.65 (0.559 to 0.742)	1.21	44.4 (31.5 to 57.4)	83.8 (77.5 to 89.4)
fPSA/tPSA ratio	20.7 [15.1; 27.9] (3.8 to 44.3)	12.8 [9.5; 17.1] (4 to 41.9)	<0.001	0.757 (0.679 to 0.836)	15.97	74.1 (63 to 85.2)	69 (61.3 to 76.8)
p2PSA (pg/mL)	14.8 [10.9; 19.3] (1.3 to 29.1)	18.4 [13.9; 27.9] (6.1 to 55.2)	<0.001	0.687 (0.607 to 0.768)	16.4	68.5 (55.6 to 79.6)	64.8 (57 to 72.5)
p2PSA/fPSA ratio	0.75 [0.56; 0.88] (0.19 to 1.85)	1.48 [1.1; 2.1] (0.57 to 4.64)	<0.001	0.935 (0.898 to 0.972)	0.93	87 (77.8 to 96.3)	91.5 (86.6 to 95.8)
PHI	31.6 [27.5; 36.8] (12.7 to 46.3)	58.6 [48.5; 76.4] (25.9 to 179.3)	<0.001	0.969 (0.948 to 0.99)	42.7	96.3 (90.7 to 100)	90.8 (85.9 to 95.1)
PHI density	0.48 [0.38; 0.67] (0.18 to 1.89)	1.36 [0.89; 1.99] (0.25 to 6.61)	<0.001	0.889 (0.835 to 0.944)	0.781	85.2 (75.9 to 94.4)	81.7 (74.6 to 88)

PSA, prostate specific antigen; fPSA/tPSA, free PSA/total PSA; p2PSA, [−2]proPSA; PI-RADS, Prostate Imaging Reporting and Data System; PHI, prostate health index; AUC, area under curve; PCa, prostate cancer; ROC, receiver operating characteristic.

**Table 3:** Demographic and clinical characteristics of the study population stratified by presence of csPCa at RP. For each variable, the AUC of the corresponding ROC curve with the 95% confidence interval (CI) is shown. Best threshold for the identification of csPCa, according to the maximization of the Youden index, is reported with the accomplished sensitivity and specificity. Cases are defined as subjects with values equal to or greater than threshold, except for prostate volume where the opposite applies.

Studied Variables	No csPCa (*n* = 26; 22.0%)	csPCa (*n* = 90; 78.0%)	*p*-Value	AUC (95% CI)	Best Threshold	Specificity (95% CI)	Sensitivity (95% CI)
Age	64.3 ± 7.3 (48 to 79)	65.9 ± 6.8 (49 to 81)	0.311	-			
PI-RADS score	3.5 [2; 4] (2 to 5)	4 [4; 5] (2 to 5)	<0.001	0.709 (0.593 to 0.825)	4	50 (30.8 to 69.2)	85.6 (77.8 to 92.2)
Prostate volume (mL)	47 [26.5; 80] (12 to 95)	46 [33; 61.2] (14 to 120)	0.61	0.467 (0.314 to 0.62)	77.5	26.9 (11.5 to 46.2)	88.9 (82.2 to 94.4)
PSA (ng/mL)	6.1 ± 1.7 (3.1 to 9.1)	6.6 ± 2.1 (2.6 to 10.4)	0.197	0.568 (0.451 to 0.685)	6.6	73.1 (53.8 to 88.5)	52.2 (42.2 to 62.2)
PSA density	13.54 [7.1; 18.46] (4 to 76)	12.97 [9; 21.32] (5 to 32)	0.596	0.588 (0.448 to 0.728)	12.3	38.5 (19.2 to 57.7)	90 (83.3 to 95.6)
fPSA (ng/mL)	0.95 [0.55; 1.4] (0.17 to 2.11)	0.7 [0.56; 1.12] (0.32 to 2.27)	0.171	0.466 (0.329 to 0.602)	1.33	61.5 (42.3 to 80.8)	48.9 (38.9 to 58.9)
fPSA/tPSA ratio	15.1 [9.5; 25.7] (3.8 to 34.6)	12.5 [7.9; 16.6] (4 to 26.1)	0.061	0.621 (0.485 to 0.758)	14.9	26.9 (11.5 to 46.2)	97.8 (94.4 to 100)
p2PSA (pg/mL)	17.7 [12.0; 27.8] (3.3 to 38.5)	18.4 [13.9; 28.9] (8.4 to 55.2)	0.585	0.535 (0.4 to 0.671)	10.7	23.1 (7.7 to 42.3)	94.4 (88.9 to 98.9)
p2PSA/fPSA ratio	1.28 [0.88; 1.49] (0.7 to 2.29)	1.73 [1.17; 2.23] (0.83 to 4.64)	0.001	0.72 (0.614 to 0.826)	1.56	80.8 (65.4 to 92.3)	56.7 (46.7 to 66.7)
PHI	48.3 [39.3; 60.9] (30.7 to 87.9)	66.3 [51.9; 81.2] (34.2 to 179.3)	<0.001	0.747 (0.637 to 0.857)	61.68	88.5 (76.9 to 100)	53.3 (43.3 to 63.3)
PHI density	1.26 [0.6; 1.67] (0.39 to 5.07)	1.45 [0.94; 2.05] (0.54 to 5.97)	0.161	0.591 (0.45 to 0.731)	0.66	38.5 (19.2 to 57.7)	93.3 (87.8 to 97.8)

PSA, prostate-specific antigen; fPSA/tPSA, free PSA/total PSA; p2PSA, [−2]proPSA; PI-RADS, Prostate Imaging Reporting and Data System; PHI, prostate health index; AUC, area under curve; csPCa, clinically significant prostate cancer; RP, radical prostatectomy; ROC, receiver operating characteristic.

**Table 4:** Multivariable logistic regression models for the identification of csPCa at RP. Results of the models are expressed as odds ratios with the corresponding 95% confidence intervals.

Variables in the Model	Base Model	Base Model + PHI	Base Model + PI-RADS
Age	1.06 (0.99 to 1.14); *p* = 0.112	1.06 (0.98 to 1.14); *p* = 0.158	1.04 (0.97 to 1.13); *p* = 0.276
PSA	1.05 (0.82 to 1.33); *p* = 0.71	0.92 (0.71 to 1.2); *p* = 0.554	1.07 (0.81 to 1.4); *p* = 0.65
fPSA/tPSA	0.9 (0.83 to 0.97); *p* = 0.009	0.94 (0.87 to 1.02); *p* = 0.166	0.88 (0.81 to 0.97); *p* = 0.007
Prostate volume	1 (0.98 to 1.02); *p* = 0.833	1.01 (0.99 to 1.03); *p* = 0.492	1.01 (0.99 to 1.04); *p* = 0.333
PHI	-	1.06 (1.02 to 1.1); *p* = 0.004	-
PI-RADS score	-	-	3.03 (1.7 to 5.42); *p* < 0.001
AUC (95% CI)	0.680 (0.557 to 0.803)	0.784 (0.676 to 0.891)	0.786 (0.66 to 0.912)
*p* for AUC comparison	ref	0.045	0.026

PSA, prostate-specific antigen; fPSA/tPSA, free PSA/total PSA; PI-RADS, Prostate Imaging Reporting and Data System; PHI, prostate health index; AUC, area under curve; mpMRI, multiparametric magnetic resonance; csPCa, clinically significant prostate cancer; RP, radical prostatectomy; ROC, receiver operating characteristic; ref, reference.

## Data Availability

The datasets used and/or analyzed during the current study are available from the corresponding author on reasonable request.

## Abbreviations

PHI: prostate health index; fPSA: free PSA; PCa: prostate cancer; PSA: prostate-specific antigen, pPSA: proPSA; p2PSA: [−2]proPSA; DRE: digital rectal examination; CIs: confidence intervals; AUC: area under curve; PI-RADS v2: Prostate Imaging Reporting and Data System version 2.
